# Candidal Retinitis in a Gynecologic Patient

**DOI:** 10.1155/S106474499500007X

**Published:** 1995

**Authors:** Pleas R. Copas, Cyril O. Spann, Jennifer I. Lim, Ira R. Horowitz

**Affiliations:** 1 Department of Gynecology and Obstetrics Emory University School of Medicine 1639 Pierce Drive P.O. Box 21246 Atlanta GA 30322 USA; 2 Department of Ophthalmology Emory University School of Medicine Atlanta GA USA

## Abstract

*Background:* Candidal retinitis is a rare but potentially devastating infection in the postoperative patient. Due to the possibility of blindness if the diagnosis and treatment are delayed, we present this report to help educate gynecologic surgeons.

*Case:* A postmenopausal patient presented for the treatment of ovarian carcinoma. Her surgical therapy required radical tumor debulking with partial bowel resection. The patient was begun on intravenous (IV) hyperalimentation through a central venous catheter. On the 7th postoperative day, a cephalosporin antibiotic was administered. Because of persistent fever, a septic workup was instituted and revealed an infected central venous catheter that was culture positive for *Candida albicans*. The patient complained of visual disturbances and an ophthalmological examination revealed candidal retinitis. Amphotericin B and fluconazole were administered with resolution of her fever and visual changes.

*Conclusion:* The risk factors of malignancy, abdominopelvic surgery, antibiotic therapy, and IV catheters are discussed. In view of the common association of these iatrogenic factors in gynecologic and obstetrical practice, we present this case to help make physicians aware of this potentially devastating infection.


Infectious Diseases in Obstetrics and Gynecology 2:228–230 (1995)
© 1995 Wiley-Liss, Inc.

### Candidal Retinitis in a Gynecologic Patient

**Pleas R. Copas, Cyril O. Spann, Jennifer I. Lim, and Ira R. Horowitz**

*Departments of Gynecology and Obstetrics (P.R.C., C.O.S., I.R.H.) and Ophthalmology (J.I.L.), Emory University School of Medicine, Atlanta, GA*

ABSTRACT

***Background:*** **Candidal retinitis is a rare but potentially devastating infection in the postoperative patient. Due to the possibility of blindness if the diagnosis and treatment are delayed, we present this report to help educate gynecologic surgeons.**

***Case:*** **A postmenopausal patient presented for the treatment of ovarian carcinoma. Her surgical therapy required radical tumor debulking with partial bowel resection. The patient was begun on intravenous (IV) hyperalimentation through a central venous catheter. On the 7th postoperative day, a cephalosporin antibiotic was administered. Because of persistent fever, a septic workup was instituted and revealed an infected central venous catheter that was culture positive for *Candida albicans*. The patient complained of visual disturbances and an ophthalmological examination revealed candidal retinitis. Amphotericin B and fluconazole were administered with resolution of her fever and visual changes.**

***Conclusion:*** **The risk factors of malignancy, abdominopelvic surgery, antibiotic therapy, and IV catheters are discussed. In view of the common association of these iatrogenic factors in gynecologic and obstetrical practice, we present this case to help make physicians aware of this potentially devastating infection.** © 1995 Wiley-Liss, Inc.

KEY WORDS

*Candida albicans*, sepsis, candidemia

Although Miale[1] described a histologically documented case of hematogenous candidal endophthalmitis in 1943, this infectious entity remains an uncommon occurrence. Due to the possible devastating disability of blindness if a delay in diagnosis occurs, physicians should become aware of the presentation, diagnosis, etiology, and treatment of this infectious disease. Since iatrogenic factors of antibiotic therapy, abdominopelvic surgery, and usage of intravenous (IV) catheters are commonly present in the gynecologic and obstetrical patient, we present a case occurring in a postoperative patient and discuss the literature to educate physicians caring for female patients.[2]

### CASE REPORT

A.H. is a 59-year-old white female who presented to the Gynecologic Oncology Section for a presumptive diagnosis of ovarian carcinoma. The patient underwent an exploratory laparotomy with tumor debulking including a partial colectomy. The patient was begun on IV hyperalimentation through a central venous catheter. On the 7th postoperative day, a urinary-tract infection was diagnosed and a cephalosporin antibiotic was administered. Cultures from the catheter and peripheral blood during the evaluation of persistent fever grew *Candida albicans*. The central catheter was removed and the patient was placed on amphotericin B. She was switched to oral fluconazole due to rising creatinine levels. Three days after candidemia was detected, the patient began to complain of black spots before her eyes. An ophthalmologic consultation was obtained and a diagnosis of candidal retinitis made. A funduscopic examination revealed the characteristic multiple white-to-yellow spots of candida (Figs.

Address correspondence/reprint requests to Dr. Ira R. Horowitz, Department of Gynecology and Obstetrics, Emory University School of Medicine, 1639 Pierce Drive, P.O. Box 21246, Atlanta, GA 30322.

Received April 18, 1994
Accepted September 30, 1994

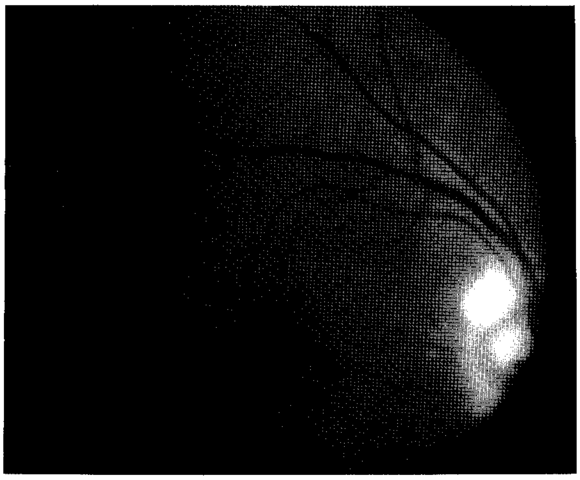

Fig. 1. Fundus photograph of the patient's right eye showing a discrete area of intraretinal whitening consistent with fungal retinitis. There are also areas of subretinal hemorrhage. There is marked myelination of the nerve fiber layer around the optic nerve head.

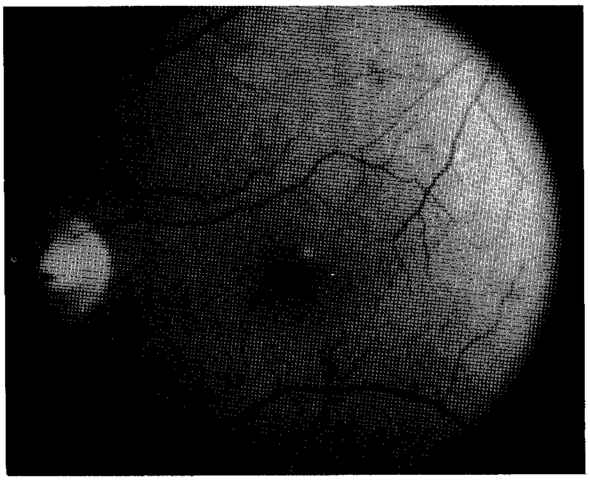

Fig. 2. Fundus photograph of the left eye showing a focal area of fungal retinitis within the macular region. There are other areas of fungal retinitis and hemorrhage noted on the fundus.

1-2). With continued treatment, the visual changes regressed and, through 6 months of follow-up, no further signs of candidemia or retinitis have been detected.

### DISCUSSION

The presenting complaint of the patient with candidal retinitis is usually a change in vision that may be reported as black spots, a veil across the eye, blurred vision, or blindness.[3] Obviously, the patient may have no symptoms or be moribund and unable to relay her complaints.[3] Two-thirds of such patients have bilateral disease and over one-half have vitreous involvement by the time symptoms develop.[2,4] Eighty-two percent of patients with disseminated candidal infections have a chorioretinal locus among the sites of the disease. A search for retinal involvement should be undertaken in all patients suffering from candidal septicemia.[5–7] The ophthalmoscopic findings of nondiscrete, fluffy, yellow lesions, which develop satellite areas and vitreous involvement as a late feature, are hallmarks of this condition.[7,8]

Our patient serves to represent many of the high-risk features for candidemia and retinitis. Initially, she presented with widespread carcinoma, a feature noted in previous reports.[3,9,10] The initial therapy of radical surgical debulking that necessitated bowel resection has been shown to be a predisposing factor.[10,11] The association of antibiotics and candidal infections has become apparent with the widespread usage of antibiotics for urinary-tract infections.[12,13] Another major predisposing factor in this case was the usage of parenteral hyperalimentation and a long-term indwelling catheter.[10,11,14]

Even though our patient was not shown to have an altered immune status, many believe that patients with overwhelming carcinoma exist in such a state.[15] The association of candidal retinitis with acquired immunodeficiency syndrome (AIDS) and congenital immune deficiencies has been reported.[7,8] The treatment of candidemia and associated retinitis has changed significantly with the introduction of newer antifungals.

The treatment in earlier reports was limited to nystatin or amphotericin B.[2,3] More recent reports have noted the successful introduction of miconazole and ketoconazole for fungal retinitis.[10,16] Our patient was successfully treated with a combination of amphotericin B and the newer agent fluconazole.

Although *C. albicans* is the most common fungus species to cause retinitis and has been addressed recently in the ophthalmologic literature, we failed to find a report in gynecologic or obstetrical journals.[9,10] We present this case occurring in a gynecologic patient to make physicians caring for the female patient aware of this rare and potentially devastating infection.

### REFERENCES

1. Miale JB: *Candida albicans* infection confused with tuberculosis. Arch Pathol 35:427, 1943.
2. Edwards JE, Foos RY, Montgomerie JZ, Guze LB: Ocular manifestations of candida septicemia: Review of seventy-six cases of hematogenous candida endophthalmitis. Medicine 53:47, 1974.
3. Van Buren JM: Septic retinitis due to *Candida albicans*. AMA Arch Pathol 65:137, 1958.
4. Griffin JR, Pettit TH, Fishman LS, Foos RY: Blood-borne candida endophthalmitis: A clinical and pathologic study of 21 cases. Arch Ophthalmol 89:450, 1973.
5. Griffin JR, Foos RY, Pettit TH: Relationship between candida endophthalmitis, candidemia, and disseminated candidiasis. In Concilium Ophthalmologicum. Amsterdam: Excerpta Medica, 1971.
6. Parke DW, Jones DB, Gemtry LO: Endogenous endophthalmitis among patients with candidemia. Ophthalmology 89:789, 1982.
7. Chess J, Kaplan S, Rubinstein A, Wang F, Wiznia A: Candida retinitis in bare symphocyte syndrome. Ophthalmology 93:696, 1989.
8. Schuman JS, Friedman AH: Retinal manifestations of the acquired immune deficiency syndrome (AIDS): Cytomegalovirus, *Candida albicans, Cryptococcus,* toxoplasmosis, and *Pneumocystis carinii*. Trans Ophthalmol Soc UK 103: 177, 1983.
9. Pesin SR, Thomas MA, Smith ME: Combined rhegmatogenous-traction retinal detachment following successful treatment of candida chorioretinitis. Arch Ophthalmol 110:1051, 1992.
10. Arocker-Mettinger E, Kramsall P, Stur M, Grabner G: Candida-retinitis following gastrectomy. Klin Monatsbl Augenheilkd 191:53, 1987.
11. Fleming KO: *Candida albicans* abscess of retina. Can J Ophthalmol 7:132, 1972.
12. Zimmerman LE: *Candida* and *Aspergillus* endocarditis with comments on role of antibiotics in dissemination of fungus disease. Arch Pathol 50:591, 1950.
13. Woods JWE, Manning IH, Patterson CN: Monilial infections complicating therapeutic use of antibiotics. JAMA 145:2087, 1951.
14. Rodrigues RJ, Shinya H, Wolff WI, Puttlitz DL: *Torulopsis glabrata* fungemia during prolonged intravenous alimentation therapy. N Engl J Med 284:549, 1971.
15. DiSaia PJ: Studies in cell-mediated immunity in two gynecologic malignancies. Cancer Bull 23:65, 1971.
16. Fitzsimons RB, Nicholls MD, Billson FA, Robertson TI, Hersey P: Fungal retinitis: A case of *Torulopsis glabrata* infection treated with miconazole. Br J Ophthalmol 64:672, 1980.
17. McDonald HR, Debustros S, Sipperly JO: Vitrectomy for epiretinal membrane with candida chorioretinitis. Ophthalmology 97:466, 1990.
